# Supplementary figures and images for: Analysis of blood parameters and molecular endometrial markers during early reperfusion in two ovine models of uterus transplantation
Source: PLoS One. 2021 May 18;16(5):e0251474. doi: 10.1371/journal.pone.0251474 (PMC8130915; doi:10.1371/journal.pone.0251474)

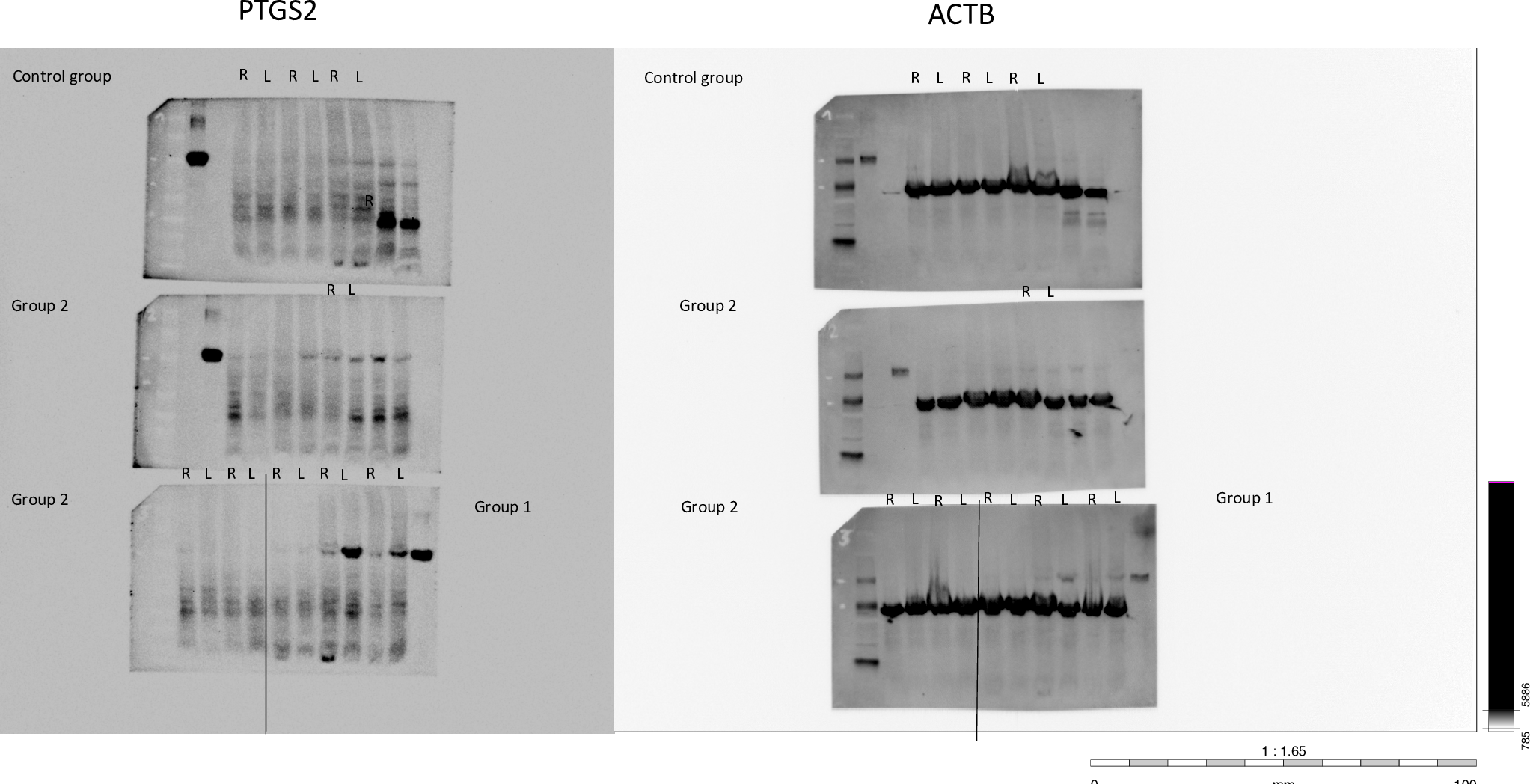

Supplement: S1 Fig — R: right horn, L: left horn. (TIF) [file pone.0251474.s001.tif]
