# Supplementary material for: Analysis of blood parameters and molecular endometrial markers during early reperfusion in two ovine models of uterus transplantation
Source: PLoS One. 2021 May 18;16(5):e0251474. doi: 10.1371/journal.pone.0251474 (PMC8130915; doi:10.1371/journal.pone.0251474)
Supplement: Attachment — Submitted filename: Carbonnel_RebuttalLetter_PLOSONE_ReluOS_26mars2021.docx [file pone.0251474.s002.docx]

**REBUTTAL LETTER**

**Responses to Reviewer #1**

***Overall, the MS would be greatly strengthened by a thorough editing for English grammar, sentence structure, and tenses of verbs etc. Some sentences are difficult to understand as written. For example, to name a few, lines 375 -376; Lines 473-474 (what is meant by a high-quality endometrium?) Lines 480-481.***

*The manuscript was edited by a fluent English speaker.*

- We clarified some sentences. For example: LINE 356-358. “Nevertheless, PTGS2 labelling intensity appears to be the most intense in the endometrium of control group compared with group 1 and 2, and the lowest in the endometrial tissue sampled from group 2 uteri (Fig 8).”459-460. “Receptive and synchronized endometrium is necessary for implantation and pregnancy [55]”Lines 466-468 “Detection of PTGS2 signal in endometrial cells in this group may be a consequence of progesterone released by the vaginal pessary used to homogenize progesterone levels between ewes before surgery[35].”

***In the introduction a brief narrative on the rationale and justification for uterine transplants would reinforce the significance of the MS. Currently, it is not clear why transplants are needed or performed. How common is this?***

- We added in the introduction the rationale and justification for uterine transplantation: “In vitro fertilization (IVF), developed over 40 years ago for treating tubal infertility, today is used to bypass nearly all infertility issues. However, one cause of infertility remains untreated: uterine factor infertility (UFI). UFI, which is estimated to affect 1-5% of women, can be congenital (complete uterine agenesis or other severe congenital malformations) or acquired (from hysterectomy or loss of a functional endometrium)[1]”. Line 41-44.

***For the western blots assay, how much protein was loaded per well? Please include.***

Eleven (11) µg protein were loaded per well. This precision has been added in the “Material and Methods” section (LINE 210).

***How was neutrophil density quantitated? Please describe.***

The density of neutrophils was quantified by counting tissue bound neutrophils in 10 area that were randomly chosen (x40 magnification), with the use of a grid. This description of the method has been added in the “Material and Method” section (LINE 158-160)

***The figure legends appear out of place in the MS and it was very difficult to ascribe specific legends to the appropriate figure when reviewing. Further all the figures are very blurry, even when the JPEG pictures were downloaded. It was difficult to read figure axis and labels etc. for all. Figure 4 needs thorough editing as data are not legible or understandable as presented.***

We apologize for any inconvenience in reading our data. The quality of figures and graphs has been improved in the revised version of our manuscript. Legends have been ascribed to the figures.
